# Genome-wide identification and expression analysis of WRKY gene family members in red clover (*Trifolium pratense* L.)

**DOI:** 10.3389/fpls.2023.1289507

**Published:** 2023-12-07

**Authors:** Guoxin Yuan, Nijing Zhang, Yiming Zou, Yaqi Hao, Jiahao Pan, Yongzhao Liu, Weiguo Zhang, Beibei Li

**Affiliations:** Key Laboratory of Resource Biology and Biotechnology in Western China, Ministry of Education, Northwest University, Xi’an, China

**Keywords:** *Trifolium pratense*, gene family, WRKY, expression patterns, abiotic stress

## Abstract

*Trifolium pratense* is an important legume forage grass and a key component of sustainable livestock development. Serving as an essential component, the *WRKY* gene family, a crucial group of regulatory transcription factors in plants, holds significant importance in their response to abiotic stresses. However, there has been no systematic analysis conducted on the *WRKY* gene family in *Trifolium pratense*. This study conducted a comprehensive genomic characterization of the *WRKY* gene family in *Trifolium pratense*, utilizing the latest genomic data, resulting in the identification of 59 *TpWRKY* genes. Based on their structural features, phylogenetic characteristics, and conserved motif composition, the WRKY proteins were classified into three groups, with group II further subdivided into five subgroups (II-a, II-b, II-c, II-d, and II-e). The majority of the TpWRKYs in a group share a similar structure and motif composition. Intra-group syntenic analysis revealed eight pairs of duplicate segments. The expression patterns of 59 *TpWRKY* genes in roots, stems, leaves, and flowers were examined by analyzing RNA-seq data. The expression of 12 *TpWRKY* genes under drought, low-temperature (4°C), methyl jasmonate (MeJA) and abscisic acid (ABA) stresses was analyzed by RT-qPCR. The findings indicated that *TpWRKY46* was highly induced by drought stress, and *TpWRKY26* and *TpWRKY41* were significantly induced by low temperature stress. In addition, *TpWRKY29* and *TpWRKY36* were greatly induced by MeJA stress treatment, and *TpWRKY17* was significantly upregulated by ABA stress treatment. In this research, we identified and comprehensively analyzed the structural features of the *WRKY* gene family in *T.pratense*, along with determined the possible roles of *WRKY* candidate genes in abiotic stress. These discoveries deepen our understandings of how WRKY transcription factors contribute to species evolution and functional divergence, laying a solid molecular foundation for future exploration and study of stress resistance mechanisms in *T.pratense*.

## 1 Introduction

Plants thrive in dynamic environments characterized by frequent unfavorable conditions that hinder their growth and development. The range of adverse environmental conditions encompasses both biotic stresses, such as pathogen infestation and herbivore predation, and abiotic stresses, including drought, high temperature, cold, UV radiation, as well as elevated levels of salt, and soil contaminants like Pd, and Cd (Zhu, 2016). The geographical distribution of plants in nature, agricultural productivity, and the sustainability of agriculture are profoundly impacted by the primary environmental factors of drought, salinity, and temperature stress. These factors impose limitations and pose threats that directly affect plants. The impact of climate change intensifies the detrimental consequences of these abiotic stresses, contributing to a rise in the occurrence of extreme weather events (Fedoroff et al., 2010). Understanding how plants perceive stress signals and acclimate to hostile environments is a fundamental inquiry in the field of biology. Enhancing plant stress tolerance is of paramount importance for both agricultural productivity and environmental sustainability, as crops with limited resilience tend to require excessive water and fertilizers, placing an immense strain on the environment.

Transcription factors (TFs) have a vital function as essential components of plant signaling pathways, actively in responses to multiple stress responses, while also coordinating internal signals in response to development and various interactions (Joshi et al., 2016). Stress-related genes are predominantly induced at the transcriptional level, and the regulation of specific stress genes’ spatiotemporal expression patterns forms a vital component of the plant’s stress response. The majority of plant’s genomic capacity is dedicated to transcription, with the *Arabidopsis* genome alone containing over 1500 encoded transcription factors (Riechmann et al., 2000). Frequently, these transcription factors are members of expansive gene families, and in certain instances, they exhibit exclusivity to the plant kingdom. When plants perceive biotic and abiotic stress, plants can spontaneously initiate a cascade of response mechanisms, including recoding of stress resistance genes, reconstruction of metabolic pathways and remodeling of cellular tissues. Stress-related TFs, upon receiving stress signals, are activated and function as molecular switches. One of the key components of this network is the interaction of stress-related transcription factors with cis-regulatory elements present in gene promoters. Through this interaction, they exert precise control over the expression of their target genes (Singh et al., 2002). There are many stress-related transcription factors in plants, such as ERF, TCP, NAC, and WRKY. These transcription factors actively contribute to the plant’s ability to cope with and respond effectively to both biotic and abiotic stresses. Among the essential and extensive families of transcription factors in plants, WRKY stands out as one of the most significant (Eulgem et al., 2000). Its role is pivotal in numerous metabolic regulatory processes, exerting a profound influence on various aspects of plant physiology and development (Rushton et al., 2010).

The structural domain of the WRKY protein is approximately 60 amino acids in length, with a highly conserved WRKYGQK heptapeptide present prominently at the N-terminus and a zinc finger structure retained at the C-terminus (Ulker and Somssich, 2004). Phylogenetically, the WRKY family is classified into three groups based on the number of conserved WRKY domains and zinc finger structures. The first group contains two conserved WRKY domains, the second group contains one conserved WRKY domain, and both groups I and II possess a C2-H2 type zinc finger structure. On the other hand, the third group comprises a conserved WRKY domain and a C2-HC-type zinc finger structure. The second subfamily further divides into five subgroups (IIa-IIe) based on the sequence characteristics of the DNA-binding structural domains within the WRKY proteins (Eulgem et al., 2000; Riechmann et al., 2000; Rushton et al., 2010). WRKY TFs exhibit selective recognition and binding capability to the W-box (C/T)TGAC(T/C) region in the promoter region of target genes (Ulker and Somssich, 2004; Jiang et al., 2017). This allows them to regulate gene expression at the transcriptional level, acting as either activators or repressors of gene expression depending on the specific regulatory context.

The initial discovery and isolation of the *WRKY* gene (*SPF1*) were first reported in sweet potato (Ishiguro and Nakamura, 1994). The WRKY protein plays a critical role in governing various stress responses associated with defense (Chen et al., 2012), as well as growth and development processes (Gao et al., 2020), phytohormone signaling, and pathogen triggered cellular responses in a variety of plants (Tsuda and Somssich, 2015; Xi et al., 2021). An instance of this is observed in maize, where the WRKY transcription factor *ZmWRKY79* plays a crucial role in enhancing drought tolerance by promoting the biosynthesis of abscisic acid (ABA) (Gulzar et al., 2021). *AtWRKY1* exerts a negative regulatory role in the defense response against *Pst.*DC3000 through the salicylic acid (SA) signaling pathway (Fang et al., 2021). *WRKY71* directly regulates the expression of ETHYLENE INSENSITIVE2 (EIN2) and ORESARA1 (ORE1), genes associated with the ethylene signaling pathway. *WRKY71* could directly activate *ACS2*, thereby stimulating ethylene synthesis and hastening the process of leaf senescence (Yu et al., 2021). In pepper, double W-box in *CaWRKY40* promoter mediates pathogen invasion and heat stress response (Liu et al., 2021). In summary, WRKYs are critical regulators in multiple stress responses, which further illustrates the potential function of WRKYs in enhancing plant stress resistance.

Red clover (*Trifolium pratense* L.) is a perennial herbaceous plant of the genus *Trifolium* in the family Fabaceae. One of the most important forage legumes in temperate agriculture and a key component of sustainable livestock husbandry, its beneficial attributes help reduce the environmental footprint of grassland agriculture (De Vega et al., 2015). Integrating leguminous forages into cropping systems improves soil health and fertility and deleterious effects of biotic and abiotic stresses (Wahdan et al., 2021). To further enhance its role in sustainable agriculture, there is an urgent need to improve our understandings of the genetic basis of genetic improvement such as persistence, disease resistance, and those traits that affect plant yield, quality and nutrition in order to expedite genetic improvement (De Vega et al., 2015).

However, so far, no studies have identified *WRKY* family in the red clover genome. Consequently, conducting a comprehensive investigation into the WRKY proteins of red clover will provide valuable insights into the fundamental molecular mechanisms underlying the development and stress resistance of this plant species. In this study, the *WRKY* family of red clover was comprehensively characterized and 59 *TpWRKYs* were finally identified. In addition, the phylogenetic relationship, chromosome distribution, gene structure, protein motifs, cis-acting elements, collinearity analysis within and between species, and protein interactions of TpWRKY proteins were systematically and comprehensively analyzed. Simultaneously, the expression profiles of *WRKY* gene members across various tissues and in response to abiotic stress and hormone treatments were examined using RNA-seq data or assessed through RT-qPCR assays. In conclusion, a comprehensive study of the *TpWRKYs* gene family and the expression pattern of *TpWRKYs* under abiotic stress lays the foundation for studying the functional properties and expression regulation of *TpWRKY*s in the growth, development, and stress response of red clover.

## 2 Materials and methods

### 2.1 Identification of *TpWRKYs* in *Trifolium pratense*

To screen for WRKY genes in *Trifolium pratense*, we obtained genomic data from EnsemblPlants. Download the *Arabidopsis* WRKY protein sequences from the official *Arabidopsis thaliana* website and use BLASTP to search for homologous sequences in the red clover protein database. (TAIR, https://www.arabidopsis.org/). Access the Pfam protein family database to download the Hidden Markov Model (HMM) file for the WRKY structural domain (PF03106) (Mistry et al., 2020), and then HMMER3.0 was used to query the WRKY gene in red clover. Based on the analyses conducted using Pfam, SMART, and CDD, it was determined that all these genes contain the intact WRKY structural domain. Analysis of molecular weight, gene location and isoelectric point of *TpWRKYs* using the seqinr R package. Determination of transmembrane regions using DEEP TMHMM, the latest update of the TMHMM software (DTU/DeepTMHMM – BioLib) (Hallgren et al., 2022).

### 2.2 Multiple sequence alignment and phylogenetic analysis

For multiple alignment analysis, multiple sequence alignment using MUSCLE (Edgar, 2004) for 151 sequences from 2 species of red clover (59), *Arabidopsis thaliana* (72), and results using msa R package to demonstrate structural features (Bodenhofer et al., 2015). We use IQ-TREE (Nguyen et al., 2015) maximum likelihood (ML) method to construct phylogenetic trees and automatically find the best model with 1000 bootstraps. WRKY proteins from different subfamilies of *Arabidopsis* were used as grouping markers. Evolutionary trees were beautified using the ITOL (Interactive Tree Of Life) online tool (Letunic and Bork, 2021).

### 2.3 Analysis of chromosomal localization, gene structure, motif, and cis-acting elements of TpWRKYs protein

The genome annotation file was used to obtain the chromosomal location of the *TpWRKY* genes, and this information was then visualized using MapChart (Voorrips, 2002). Using the Gene Structure Display Server (GSDS 2.0) to map the structure of the *TpWRKY* genes (Guo et al., 2007). The conserved motifs of TpWRKY proteins were identified using Multiple Em for Motif Elicitation (MEME,v5.4.1) program with the following parameters: number of repetitions-any, maximum output of 10 motifs, and the optimal motif range was set to 6-200 amino acids (Bailey et al., 2009). To explore the cis-acting elements present in the promoter region of the *TpWRKY* family, we extracted the 2k sequence upstream of each *TpWRKY* gene as the promoter region and submitted it to the PlantCARE database for cis-acting element prediction (Lescot et al., 2002). Ultimately, the analysis and visualization of cis-acting elements were carried out using GSDS and ggplot2 R package.

### 2.4 Subcellular localization, gene duplication and synteny analysis

Cell-PLoc 2.0 website tools were utilized for predicting subcellular localization (Chou and Shen, 2010). Checking for gene duplication events using the default parameters of the Multiple Collinearity Scan toolkit (MCScanX) (Wang et al., 2012). To explore covariation between red clover and other species, JCVI (Tang et al., 2015) was used to visualize covariation results for red clover with *Arabidopsis* and rice. Circos (Krzywinski et al., 2009) was used to visualise the results of intraspecific covariation in red clover.

### 2.5 Plant materials, growth conditions and treatments

Growing red clover in the plant greenhouse at Northwest University, the growing conditions are set for 16h light at 25°C and 8h dark at 22°C, with humidity at around 60%. Seedlings of about 60d of healthy growth and uniform size were then selected for subsequent experiments. In the drought treatment, seedlings in Pots were watered with 20% PEG8000. The seedlings were moved to 4°C in an incubator for low temperature stress. The above-ground surface parts of the seedlings were sprayed with 100μM MeJA and ABA for hormone stress treatment, separately. Samples were then taken at 0h (as control), 3h, 6h, 12h, 24h and 48h, respectively, and immediately frozen in liquid nitrogen after sampling at different time points, followed by storage at -80°C for subsequent experiments. Each treatment was independently replicated three times, and for each replicate, samples were collected from three different plants. In order to study the expression pattern of *TpWRKY* genes under different stress treatments, 12 *TpWRKY* genes were selected for RT-qPCR analysis.

### 2.6 RNA extraction and synthesis of cDNA

During the process of grinding each plant tissue into a fine powder, liquid nitrogen was continuously added to maintain a low temperature. For sequencing samples, total RNA was extracted from tissues using the MJZol Total RNA Extraction Kit (Majorbio, Shanghai, China). Using the *SteadyPure* Plant RNA Extraction kit (Accurate, Changsha, China), total RNA was extracted from the stress-treated samples. After 1% agarose gel electrophoresis, the integrity of the RNA was checked, and then the RNA quality was determined using a 5300 Bioanalyzer (Agilent). The RNA was quantified using an ND-2000 (NanoDrop Technologies), and high-quality samples were selected for the construction of sequencing libraries. For quantitative cDNA synthesis, reverse transcription was performed with 1μg RNA in a 10 μl system according to the instructions for the Reverse Transcription Kit AG11706 (Accurate, Changsha, China).

### 2.7 Library preparation, sequencing and RNA-Seq data analysis

At Shanghai Majorbio Bio-pharm Biotechnology Co., Ltd (Shanghai, China), RNA purification, reverse transcription, library construction, and sequencing of the samples were conducted for the sequenced samples. The paired-end RNA-seq sequencing library was finally sequenced (2 x 150 bp read length) using a NovaSeq 6000 sequencer. Quality control and filtering of the raw data from the lower machine using fastp, with clean reads compared to the reference genome by HISAT2 (Chen et al., 2018; Kim et al., 2019). Reads were counted using featureCounts in the subread package (Liao et al., 2013), then normalized by Transcripts Per Kilobase per Million mapped reads (TPM) and finally TPM values were used to assess gene expression levels. Heat map construction of *TpWRKY* gene expression profile with R. We uploaded raw data to NCBI under project number PRJNA1035639.

### 2.8 RT-qPCR analysis

RT-qPCR was carried out using SYBR Green Pro Taq HS (Accurate, Changsha, China) as the fluorescent dye. The PCR reaction conditions consisted of an initial denaturation step at 95°C for 30 seconds, followed by 40 cycles of denaturation at 95°C for 10 seconds, and annealing/extension at 60°C for 30 seconds. The expression levels of the 15 selected WRKY genes were studied under drought and cold stress as well as MeJA and ABA treatments. Three biological replicates were performed for each sample and there were also three technical replicates for each biological replicate, using a 10-fold dilution of cDNA as template, and primers are listed in the **Supplementary Table S1**. The relative expression of the *TpWRKY* gene was calculated using the 2^-ΔΔCT^ method, using ACTIN as an internal reference gene, and subjected to statistical analysis. Asterisks (*, **, ***, ****) indicate p < 0.05, 0.01, 0.001 or 0.0001, respectively, and the differences are statistically significant.

## 3 Results

### 3.1 Identification of the WRKY family in *T.pratense*

The WRKY domain model (PF03106) and the protein sequences of *Arabidopsis thaliana* WRKY proteins were utilized as search queries to identify candidate *WRKY* genes in the protein files of red clover. To eliminate redundant sequences and ensure consistent structural features, a filtering process was conducted. The remaining sequences were subjected to identification in SMART, CDD, and Pfam databases. Consequently, this analysis yielded a set of 59 putative *WRKY* genes, which were systematically named as *TpWRKY1* to *TpWRKY59* according to their respective chromosomal positions (**Supplementary Table S2**). The **Supplementary Table S3** contains sequences of *WRKY* genes, including genomic, CDS, and protein sequences. we conducted a comprehensive analysis of each TpWRKY proteins, examining their chromosomal location, protein length, isoelectric point (pI), subcellular localization, and transmembrane structural domain. The length of TpWRKY proteins is between 146 (TpWRKY57) and 1315 (TpWRKY14) amino acids, and the molecular weight is between 16.39 kDa (TpWRKY57) and 146.86 kDa (TpWRKY14). Moreover, the pIs ranged from 4.56 (TpWRKY47) to 10.7 (TpWRKY19). TpWRKYs were predicted to subcellular localized in the nucleus, except that TpWRKY48 was predicted to mitochondria and nucleus, possibly functioning in the mitochondria. All proteins have no transmembrane domain, indicating that they are non-membrane proteins. TpWRKY proteins domain signatures significantly differentiate between varieties of themselves, which suggests their functional diversities in *Trifolium pretense* L.

### 3.2 Multiple sequence alignment and phylogenetic classification of *TpWRKYs*

To elucidate the distinctive characteristics of the WRKY domain in each TpWRKY proteins, we performed multiple sequence alignment analysis on the approximately 60 amino acids that constitute the WRKY domain. For this analysis, we compared the sequences of 72 AtWRKY proteins and 59 TpWRKY proteins, as depicted in **Supplementary Figure S1**. As a result, it was observed that complete or nearly complete conserved sequences of WRKYGQK were found in 59 TpWRKY proteins. Interestingly, certain conserved motifs in some of the TpWRKY proteins exhibit specific mutations and evolutionary changes in a few amino acids (**Figure S1**). For instance, Q mutated to K in the conserved motif of WRKYGQK in TpWRKY4, TpWRKY25, TpWRKY29, TpWRKY47, Q mutated to R in TpWRKY43, TpWRKY54, TpWRKY55, Q mutated to E in TpWRKY22, and R mutated to I in TpWRKY11. Furthermore, it was observed that the gene sequences of TpWRKY34 and TpWRKY48 lacked zinc finger structures, while some other TpWRKY proteins contained either C2H2 or C2CH zinc finger domains.

**Figure 1:**
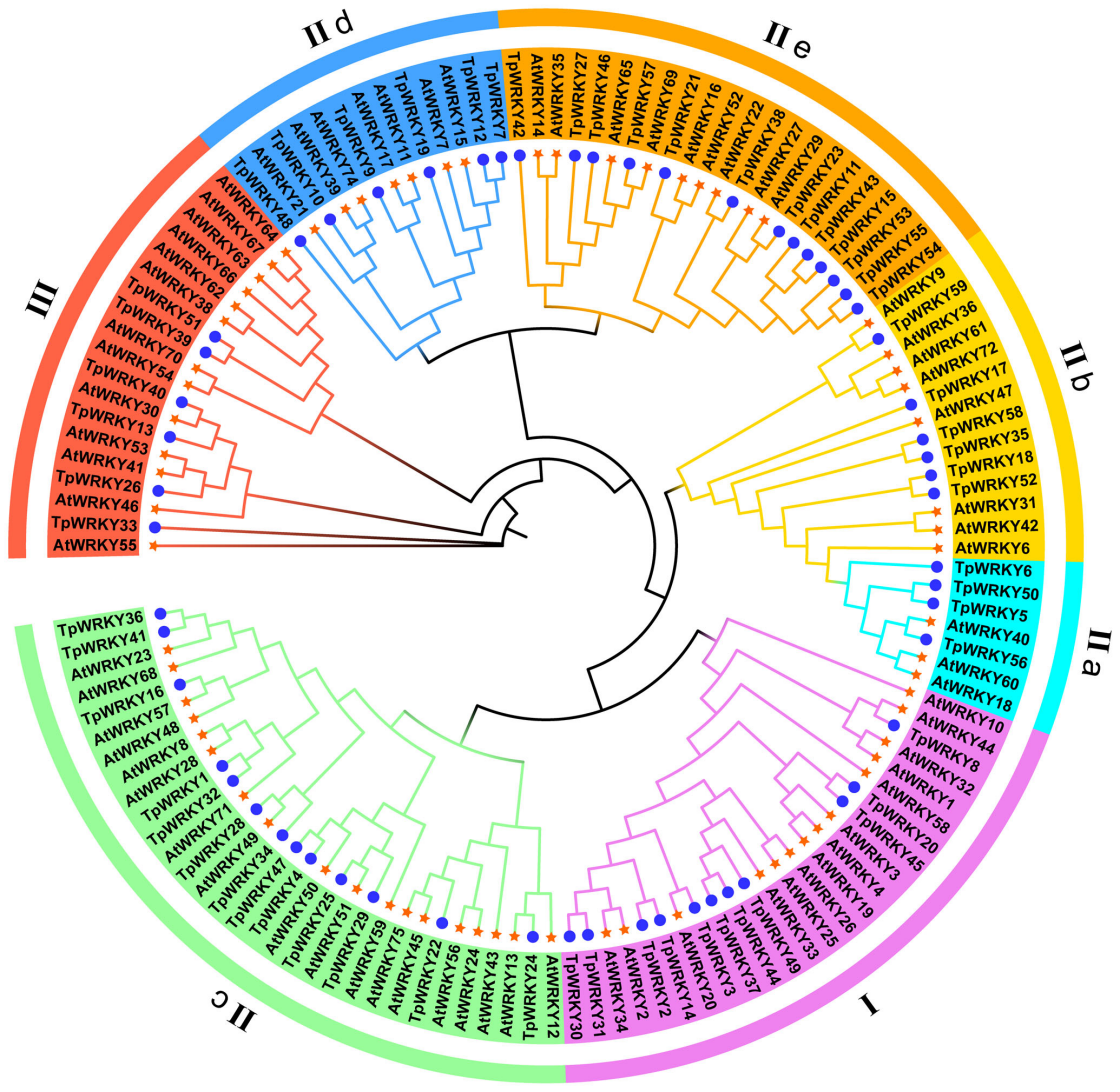
Phylogenetic analysis of the full-length structural domains of WRKY proteins in red clover and *Arabidopsis thaliana*. By employing the Maximum Likelihood method, the phylogenetic tree was established using IQ-TREE software, with the application of bootstrap testing for 1000 replicates to assess its robustness. Seven groups/subgroups are indicated by different colors located outside the circle, with blue circles representing *TpWRKYs* and orange stars representing *AtWRKYs*.

To explore the evolutionary relationships among the members of the TpWRKY family, a comparative analysis with 72 AtWRKYs from *Arabidopsis* as a reference was conducted. The phylogenetic tree was constructed using the maximum likelihood method through IQTREE software, incorporating 1000 bootstrap tests to enhance confidence in the results. The tree was based on the sequences of *T. pratense* and *Arabidopsis*, effectively illustrating the genetic relatedness between the two. As a result, all TpWRKY proteins were classified into three distinct groups: Group I, Group II (IIa-IIe) and Group III (**Figure 1**). The analysis of the phylogenetic tree revealed that Group II had the most abundant representation with a total of 42 WRKY members. Group I and Group III comprised 11 and 6 members, respectively. Among the five subgroups into which Group II was further categorized, subgroups IIc and IIe had the highest number of members, each consisting of 13 members. Subgroups IIb and IId contained six members each, while subgroup IIa had the minimum representation, with only four members. The classification of TpWRKYs has substantiated the diversity in their protein structures and suggests that different members within the subfamilies may possess distinct regulatory functions. This underscores the potential for varied roles played by TpWRKY proteins in coordinating plant responses and adaptations to various environmental cues.

### 3.3 Chromosomal location of *TpWRKYs* in the *Trifolium pratense* genome

The chromosome location map displayed that the *TpWRKY* genes were evenly distributed on the chromosomes, with 10 genes on chromosomes 1 and 2, 11 genes on chromosome 3, 6 genes on chromosome 4, 2 genes on chromosome 5. Moreover, chromosomes 6 and 7 contained 6 and 13 genes, respectively. But there are fewer genes on chromosome 5, which may be related to the size and structure of chromosome 5. Moreover, by MCScanX collinearity analysis, we found a pair of tandem repeat replicates on each of chromosomes 1, 3, and 7. Remarkably, a significant majority of *TpWRKYs*, which fall under the same subfamily in the phylogenetic tree, exhibit a scattered distribution on the same chromosome or were located on different chromosomes rather than being closely clustered on the same chromosome. Furthermore, some were distributed in close proximity on the same chromosome, but they still do not belong to tandem repeat replication. These indicated that the expansion of *TpWRKY* family is predominantly influenced by mechanisms other than tandem replication events, suggesting a minor contribution of tandem replication events to its expansion.

### 3.4 Gene structure and conserved motifs of *TpWRKYs*

**Figure 2:**
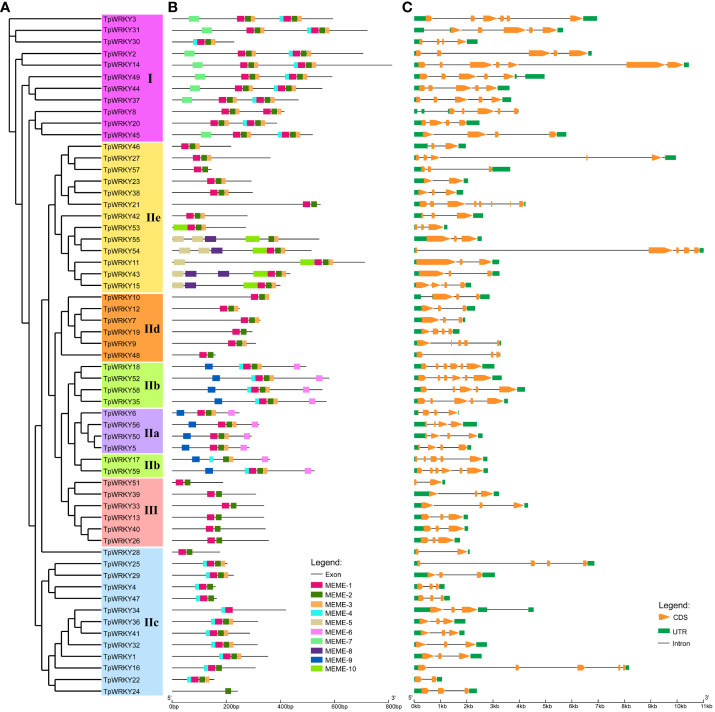
Phylogenetic tree, composition of conserved motifs and gene structures of TpWRKY family members. **(A)** The full-length structural domains of 59 TpWRKY proteins were used to construct an evolutionary tree by maximum likelihood. Different color backgrounds represent different subfamily classifications. **(B)** Conserved motifs of TpWRKY proteins. Different colors and numbers represent different motifs. **(C)** Exon/intron structure analysis of *TpWRKY* genes. Green represents the UTR region, yellow represents the exon region, and black lines represent the intron region.

To gain a comprehensive understanding of the conservation and diversity exhibited by WRKY family, we subjected all TpWRKYs to an analysis of their conserved motifs using the MEME program. Subsequently, we predicted the composition of motifs present within the TpWRKY proteins. As shown in **Supplementary Figure S2** and **Table S4**, a comprehensive selection of 10 distinct motifs was made and labeled as motif 1 to motif 10. These conserved motifs are between 15 and 51 amino acids in length. The quantity of conserved motifs for each TpWRKY proteins was primarily from 1 to 8 (**Figure 2B**). As expected, the same group or subgroup of TpWRKYs has a highly consistent conserved motif. Motif1, motif2 and motif3 are present in most genes, motif4 is present only in subgroups I, IIb and IIc, motif6 and motif9 are specific to IIa and IIb. Subgroup I contains the specific motif7 and subgroup IIe contains the specific motif5, motif8 and motif10. Notably, TpWRKY24 contains only motif 2, which may be related to the loss of the structural domain. Overall, the closely related TpWRKYs in the phylogenetic tree have similar motif compositions and may perform similar functions. However, the functions of many of these motifs are yet to be clarified.

The diversity of gene structures can provide insights into the evolutionary history of gene families. Therefore, we conducted analyzed the exon/intron distribution and the number of coding exons for each *TpWRKY* gene to further understand the evolutionary history of the WRKY family in *T. pretense*. The coding exons of *TpWRKY* genes varied in number, spanning from 2 to 8. Among the *TpWRKY* genes, the highest proportion (44%) contained 3 coding exons, followed by 5 (20%), 4 (17%), 2 (7%), 6 (8%), and 7 and 8 coding exons each (2%) (**Figure 2C**). At the same time, the number of coding exons of protein members in the same family converged, thus confirming the phylogenetic plausibility. For example, subgroup IIa TpWRKYs contained 3-4 coding exons, with an intron count of 3. Subgroup IIb TpWRKYs contained 5-6 coding exons and 4-5 introns. Subgroup III TpWRKYs contained 2-3 coding exons and 1-2 introns.

Overall, TpWRKYs that show close relationships in the phylogenetic tree tend to have similar gene structures and conserved motif compositions, suggesting that TpWRKYs within the same taxon may likely have comparable functions.

### 3.5 Analysis of cis-acting elements in the promoter region of *TpWRKY* genes

**Figure 3:**
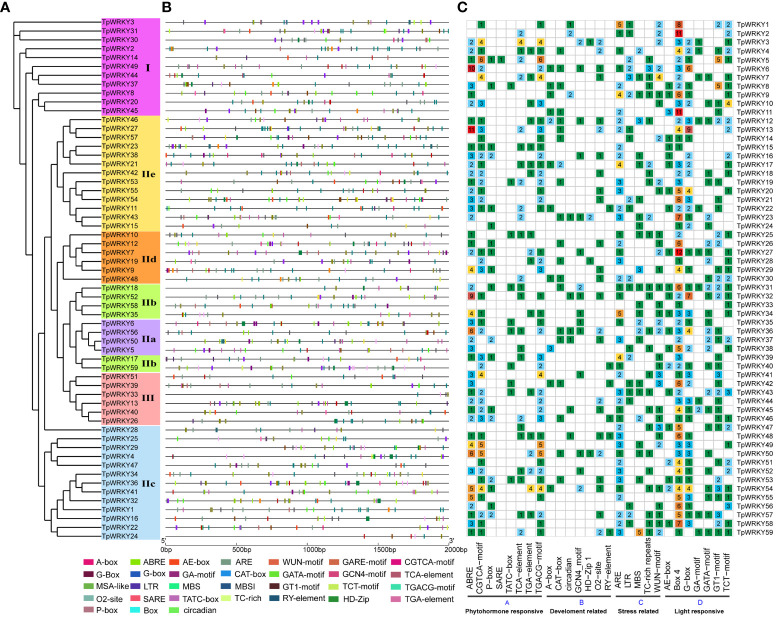
Cis-element analysis of the 2kb region upstream of the *TpWRKY* genes. **(A)** The full-length structural domains of 59 TpWRKY proteins were used to construct an evolutionary tree by maximum likelihood. Different color backgrounds represent different subfamily classifications. **(B)** Various types of cis-elements and their respective locations in each *TpWRKY* genes were depicted using distinctively colored blocks. **(C)** The grid displays colors and numbers, representing the count of different cis-acting elements in the initiation regions of *TpWRKY* genes. ggplot2 maps the type, number, and location of cis-acting progenitors of the *TpWRKY* genes.

In order to gain insights into the role of cis-regulatory elements in *TpWRKYs*, we utilized the PlantCARE software to identify cis-acting elements within the sequences located 2,000 bp upstream of the translation start site (ATG) for each *TpWRKY* genes. A total of 62 different cis-acting elements were identified in 59 *TpWRKY* genes, and we focused our analysis on cis-acting elements related to development-related, phytohormone responsive, stress-responsive, and light-responsive elements (**Figure 3** and **Supplementary Table S5**).

The first category of cis-acting elements related to plant growth and development, including related to meristem expression responsive (CAT-box), differentiation of the palisade mesophyll cells responsive (HD-Zip 1), circadian rhythm control responsive (circadian), endosperm expression responsive (GCN4_motif), metabolism regulation responsive (O2-site), seed-specific regulation responsive (RY-element). Focusing on the regulation of plant growth and development, it is worth noting that *TpWRKY6*, *TpWRKY12*, *TpWRKY23*, *TpWRKY36*, and *TpWRKY50* all exhibit the presence of four distinct plant development-related elements. They may play a key role in various aspects of plant growth and development. The second category of phytohormone-responsive cis-acting elements encompasses various elements, such as abscisic acid responsiveness (ABRE), gibberellin-responsive elements (P-box, TATC-box), MeJA-responsiveness (CGTCA-motif, TGACG-motif), auxin-responsive element (TGA-element) and salicylic acid responsiveness (TCA-element, SARE). Among them, the largest proportion of cis-acting regulatory elements was involved in ABA responsiveness and MeJA responsiveness. Of particular interest, the SARE cis-acting element is exclusively present in the promoter region of *TpWRKY5*, implying a potential involvement of this gene in the salicylic acid (SA) signaling pathway. The third category of adversity stress response cis-acting elements, including anaerobic induction responsiveness (ARE), drought-inducibility responsiveness (MBS), low-temperature responsiveness (LTR), flavonoid biosynthetic responsiveness (MBSI), wound responsiveness (WUN-motif), defense and stress responsiveness (TC-rich repeats). Furthermore, 86% of the promoter regions of 51 *TpWRKY* genes were found to contain more than two stress-related cis-acting elements. This implies that *TpWRKYs* may be involved in multiple stress responses. Among the 59 *TpWRKY* genes, most cis-acting elements were found in the fourth category, which encompasses elements related to light response, such as G-box, TCT-motif, GA-motif, and others. Except for *TpWRKY16* and *TpWRKY30*, Box 4 was found in the promoter regions of nearly all *TpWRKYs*.

### 3.6 Duplication and synteny analysis of *TpWRKY* genes

**Figure 4:**
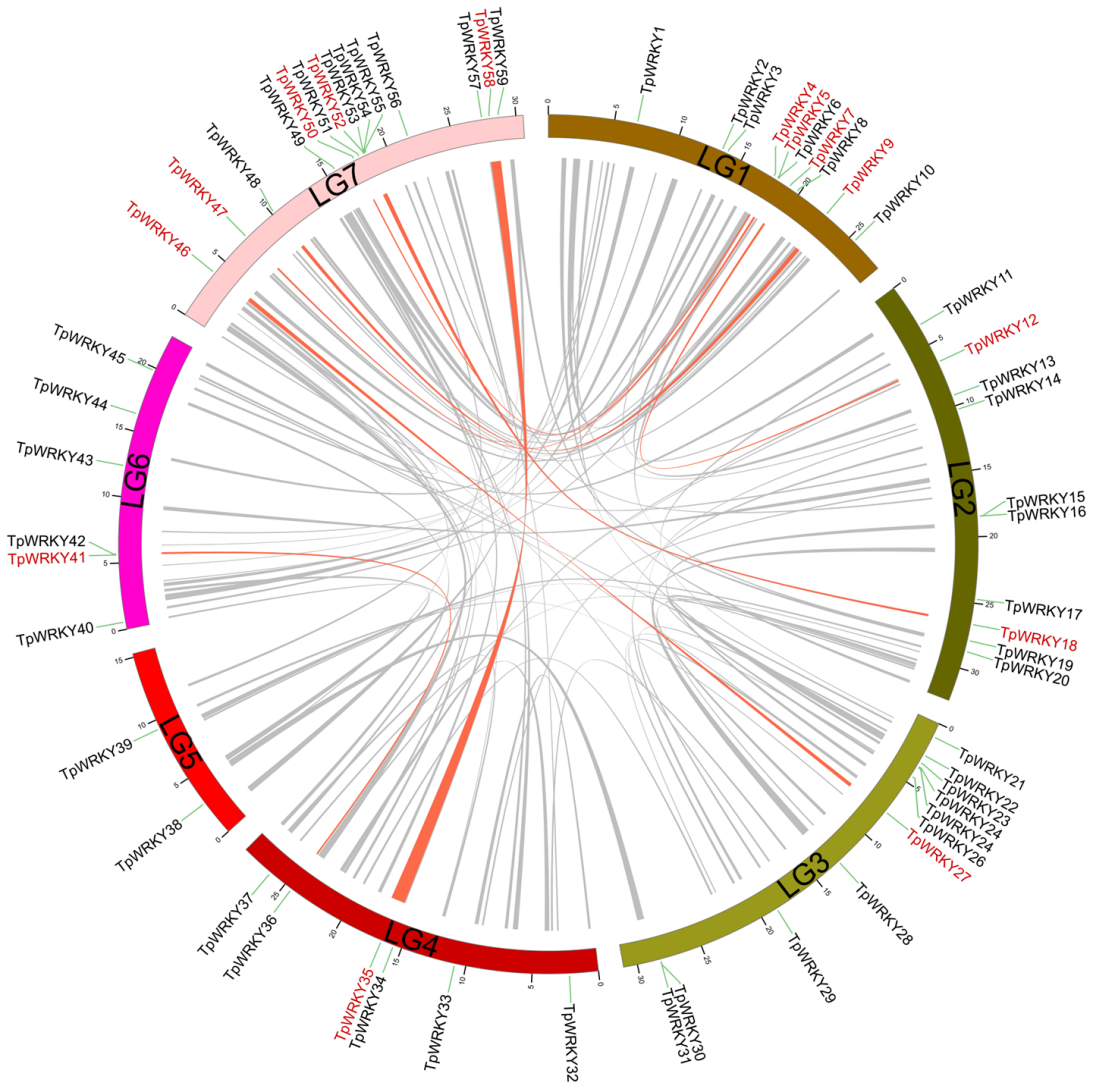
Demonstration of interchromosomal relationships in *TpWRKYs*. The gray line indicates all synteny blocks within red clover, and synteny blocks of *WRKY* genes are highlighted with red lines.

Conducting a synteny analysis of the *TpWRKY* genes using BLASTP and MCScanX aimed to explore segmental duplication events within the red clover WRKY family. We observed segmental duplication events involving 16 *WRKY* genes and found that genes forming the segmental duplication were from the same subfamily (**Figure 4**). Meanwhile, tandem duplication events, in which tandem duplication events are defined as chromosomal regions containing two or more genes within a 200 kb distance, are found in the red clover *WRKY* genes (Holub, 2001). To verify whether these gene pairs underwent purifying selection, we computed the non-synonymous substitution rate (Ka) and synonymous substitution rate (Ks). It was noticed that all homozygous *TpWRKY* gene pairs had Ka/Ks ratios below 1, indicating that these gene pairs underwent purifying selection (**Supplementary Table S6**). The results implied that specific *TpWRKY* genes could have arisen from segmental duplication events, implying that the evolution of *TpWRKY* genes is potentially influenced, at least partially, by both segmental and tandem duplication events.

**Figure 5:**
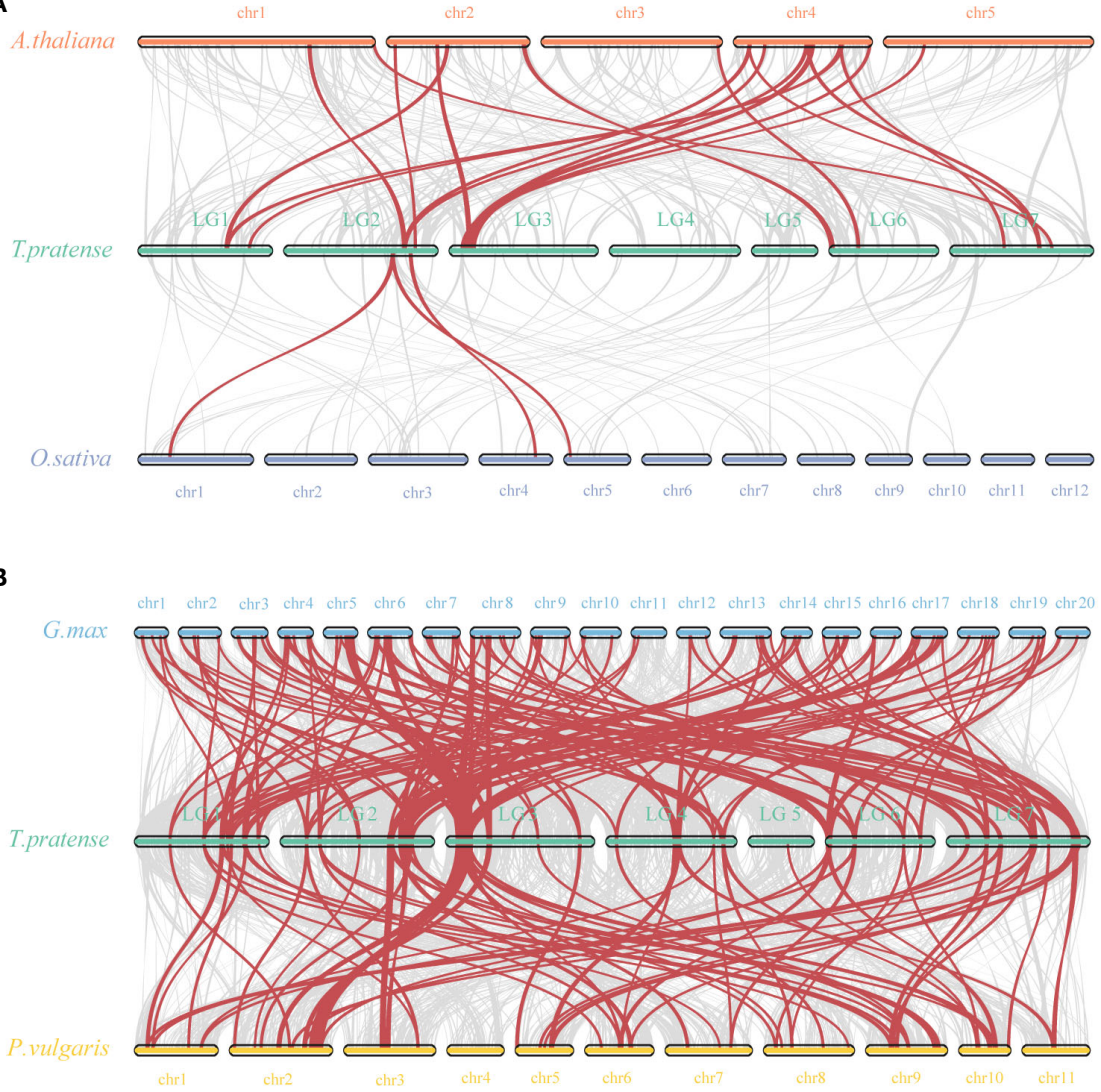
Synteny analysis of WRKYs between red clover and other plant species. **(A)** Analysis of synteny between *TpWRKY* genes and *Arabidopsis* and rice. **(B)** Analysis of synteny between TpWRKY genes and soybean and common bean. Interspecies collinear blocks are shown as gray lines, and the synteny *WRKY* gene pairs are marked in red.

To further explore the molecular evolutionary relationships between species, we used a dicotyledonous plant (*Aabidopsis*) and a monocotyledonous plant (*Oryza sativa*) to map the covariance of the red clover WRKY family (**Figure 5A**). Between red clover and *Arabidopsis*, a total of 17 collinear gene pairs were detected, and an additional 3 collinear gene pairs were found between red clover and rice. In addition, we selected soybean and common bean to further investigate the colinearity of *WRKY* genes in legumes. The colinearity analysis showed that red clover had 137 colinear gene pairs with soybean (*Glycine max*) and 70 colinear gene pairs with common bean (*Phaseolus vulgaris*) (**Figure 5B**). Based on the homology analysis of *TpWRKYs* between red clover and other species, it was indicated that the *TpWRKY* genes exhibited higher homology with legume, potentially attributable to their close genetic relationship. At the same time, they exhibit a higher homology with the dicot model plant *Arabidopsis*, indicating that these genes likely emerged after the differentiation of dicots and monocots. Despite chromosomal rearrangements or gene duplications, the synteny analysis of *TpWRKY* genes demonstrated robust collinearity.

### 3.7 *TpWRKYs* expression profiles in different organs

**Figure 6:**
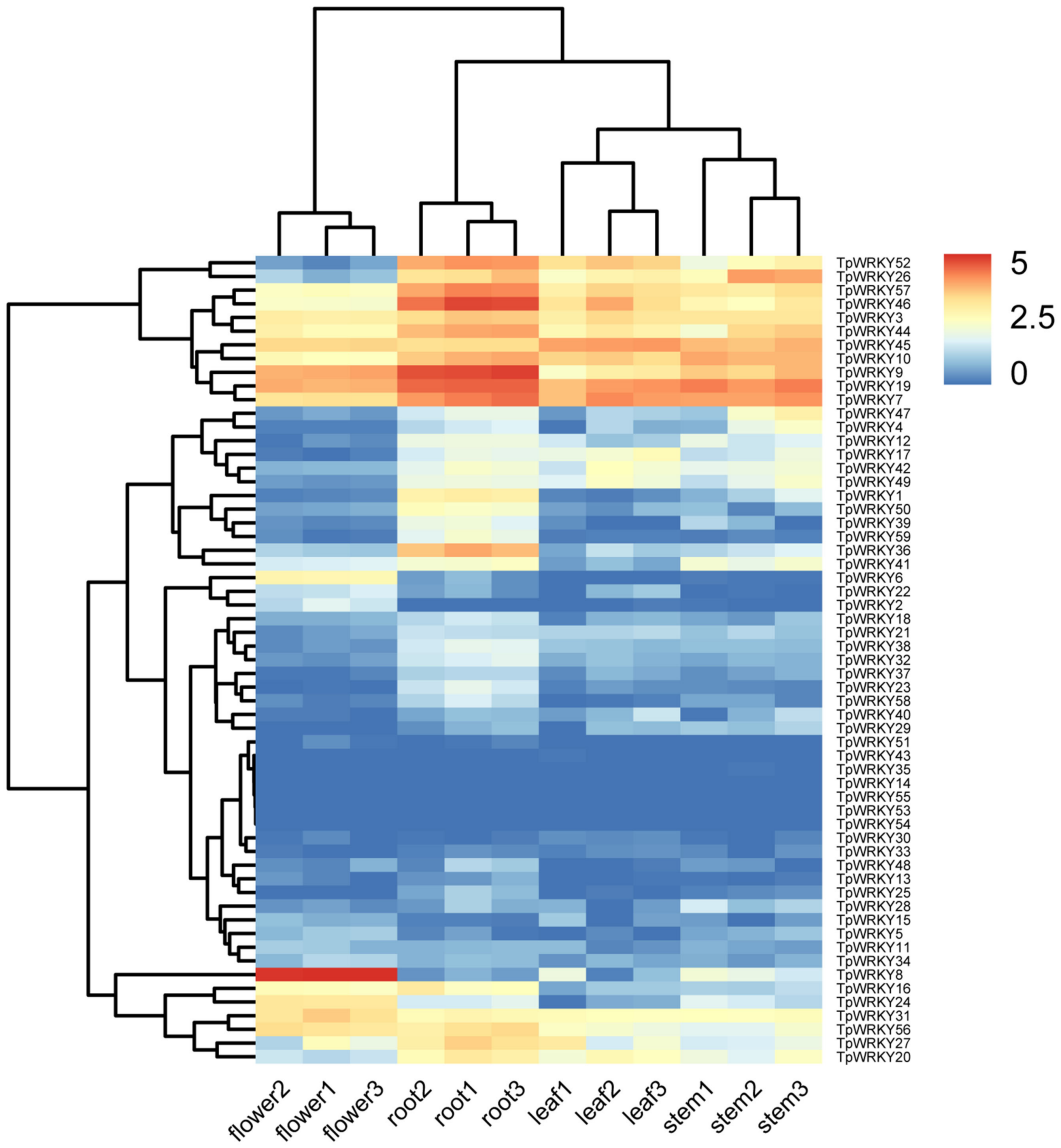
Heatmap demonstrating the expression profile of *TpWRKY* genes in four organs. *TpWRKY* gene expression heatmap was plotted using TPM values, and data were normalized relative to the average expression value of each gene in all samples and log2 transformed. Blue and red colors indicate low- and high-expressed genes, respectively.

A standard transcriptome analysis procedure was employed to investigate the transcript abundance of all 59 *TpWRKYs*, utilizing transcriptomic data from four organs of red clover, namely roots, stems, leaves, and flowers (**Figure 6** and **Supplementary Table S7**). Among the 59 *TpWRKY* genes, fifty *TpWRKY* genes were detected in four different organs (TPM > 0),17 genes were constitutively expressed in all samples (TPM > 1). No transcripts of three *TpWRKYs* (*TpWRKY53*, *TpWRKY54* and *TpWRKY55*) were detected in the different developing organs, suggesting that they may be pseudogenes. Conversely, some *TpWRKY* genes showed high expression in specific organs. For instance, 6 genes in flowers, 7 genes in leaves, 13 in roots and 9 genes in stems (TPM > 20). Highly expressed in all organs, *TpWRKY3, 7, 9, 19, 45* may serve an essential role in the overall growth and developmental process of red clover. Analysis of *WRKY* gene expression in different organs showed that *TpWRKY31* was significantly higher in flowers than in roots, stems, and leaves, whereas *TpWRKY9/36/46* had higher expression in roots and relatively lower expression in stems and leaves. Indicating their significance in numerous aspects of plant development, including both the whole plant and specific organs, these results highlighted the vital roles played by these genes.

### 3.8 Expression patterns of selected *TpWRKY* genes under hormone and abiotic treatments

In order to delve deeper into the potential impact of different stress treatments on the expression of these *TpWRKY* genes, a subset of 12 members from various subfamilies was chosen using RNA-Seq analysis, guided by the examination of known stress-related WRKY proteins and cis-acting elements. Undergoing four distinct stress treatments, red clover seedlings were exposed to drought (20% PEG8000), low temperature (4°C), ABA (100 μM), and MeJA (100 μM). Using RT-qPCR, the expression variations of the selected genes were subsequently analyzed under different stress conditions.

**Figure 7:**
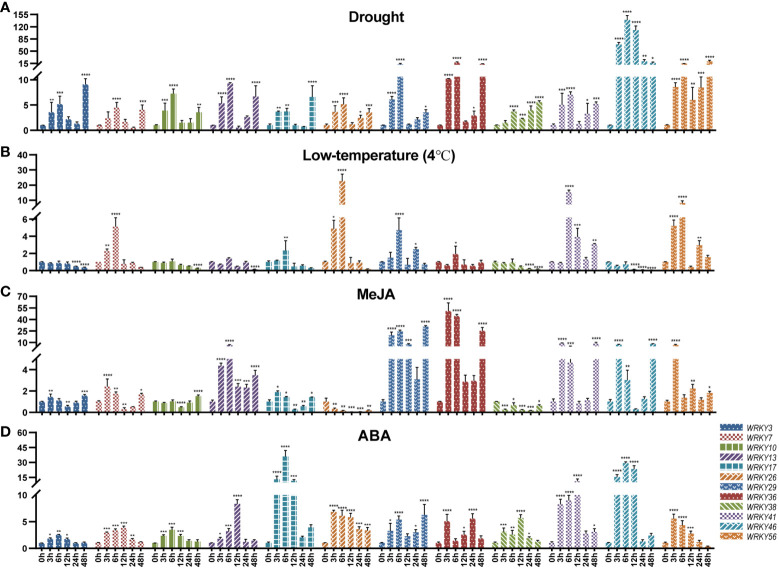
qRT-PCR analysis of 12 *TpWRKY* genes under different stress treatments at 0, 3, 6, 12, 24, and 48h. **(A)** Drought. **(B)** low temperature (4°C). **(C)** MeJA. **(D)** ABA. Non-stress level of 0h was used as control. Vertical lines show standard deviation (*n* = 3). The presence of asterisks denotes a statistically significant difference when compared to the control group. (*p < 0.05, **p < 0.01, ***p < 0.001, ****p < 0.0001).

In **Figure 7A**, the expression of all 12 genes was up-regulated under drought stress. Among them, *TpWRKY46* was highly induced after being subjected to drought stress, and its expression level peaked at 6 h 139-fold higher than that under normal conditions. It is noteworthy that with the prolongation of stress time all genes had two up-regulated peaks at 6h and 48h of drought stress, except for *TpWRKY46* gene which reached its peak at 6h of drought stress treatment. Most genes were down-regulated under low temperature stress. Among them, *TpWRKY3/10/38/46* were down-regulated at all low temperature stress treatment times, and *TpWRKY46* was most significantly down-regulated at 48h, 0.06-fold of the normal expression level. Interestingly, *TpWRKY7/17/26* were all up-regulated and then down-regulated, and all turned down-regulated at 12 h of low temperature stress treatment, with *TpWRKY26* being 22-fold up-regulated at 6 h of low temperature stress compared to normal levels (**Figure 7B**).

Under MeJA stress treatment, only *TpWRKY26/38* were down-regulated at all time periods and peaked at 24 h. *TpWRKY29/36/56* were all up-regulated, with *TpWRKY29* peaking 30-fold up-regulated at 48 h of stress treatment and *TpWRKY36* peaking 50-fold up-regulated at 3 h of stress treatment. However, the rest of the genes, except *TpWRKY10* which was down-regulated and then up-regulated, all showed an up-regulated, down-regulated and then up-regulated expression pattern (**Figure 7C**). Under ABA stress treatment, all were up-regulated except *TpWRKY56* which was up-regulated and then down-regulated. Among them, the expression of *TpWRKY3/10/17/26/46* all peaked at 6 h of ABA stress treatment, and *TpWRKY17* and *TpWRKY46* were highly induced to be expressed at 35 and 29-fold of normal levels. The expression of *TpWRKY7/13/38/41* peaked at 12 h, *TpWRKY13* and *TpWRKY41* were at normal levels of 8 and 10-fold (**Figure 7D**).

## 4 Discussion

For the past few years, more and more researchers are focusing on germplasm resources and sustainable development in grass industry (Sato et al., 2005; Yan et al., 2023). Due to its high seedling survival rate, rapid growth, and stress tolerance, red clover (*Trifolium pratense* L.) is a significant forage legume extensively cultivated in various temperate regions (Sato et al., 2005). Plant growth, development and reproduction are usually affected by a variety of biotic and abiotic stresses in natural world (Sharif et al., 2021). Plants have evolved complex regulatory mechanisms involving different TFs, which accommodate unfavorable environmental conditions (Baillo et al., 2019). *WRKY* genes are widely present in plants and play a crucial role in regulating a variety of stress responses (Jiang et al., 2017; Khoso et al., 2022). Currently, the genome-wide analysis of the *WRKY* family has been extensively conducted in numerous sequenced species (Yan et al., 2019; Hu et al., 2021; Kan et al., 2021). WRKY family in red clover has not been identified so far, which has hindered the study of the function of *TpWRKY* genes to some extent. Consequently, our study involved the identification of the WRKY family in red clover, followed by systematic bioinformatic analysis and expression analysis in response to both hormonal and abiotic stresses.

The identification of a total of 59 *TpWRKY* genes was achieved in this study, and they were named as *TpWRKY1* to *TpWRKY59* based on their chromosomal positions. There was no significant correlation between the number of *WRKY* genes and genome size. For example, *Arabidopsis thaliana* genome size 125 Mb (75), *Liriodendron chinense* genome size 1.7 G (44), and *Dendrobium catenatum* genome size 1.11 Gb (62). The quantity of red clover WRKY family members might be linked to its evolutionary process and the way of family expansion, with the quality of genome assembly also serving as a significant factor influencing the gene count. It has been shown that most of the *WRKY* genes are localized in the nucleus (Wang et al., 2018). The physicochemical property analysis revealed notable differences in the MW and pI of TpWRKY proteins, implying that their protein structures may exhibit variations, enabling them to adapt to diverse biotic and abiotic stresses. All WRKY proteins do not have transmembrane structural domains and are localized to the nucleus. These features suggest that *WRKY* genes perform different biological functions under different stresses which may be related to the physicochemical properties and spatial structure of the protein.

WRKY TFs, containing a highly conserved structural domain consisting of approximately 60 amino acids, a highly conserved WRKYGQK structural domain at the N-terminus and a variable zinc finger structure at the C-terminus. (Rushton et al., 2010). It regulates gene expression by binding specifically to the W-box in the promoter region of the target gene. At least one conserved WRKY structural domain is present in the 59 TpWRKY proteins identified in this study. Meanwhile, we found specific mutations and evolution of the highly conserved WRKYGQK core sequence of some TpWRKY proteins. Four mutations were WRKYGKK (*TpWRKY4*, *TpWRKY25*, *TpWRKY29*, *TpWRKY47*), three mutations were WRKYGRK (*TPWRKY43*, *TPWRKY54*, *TpWRKY55*), and one mutation was WRKYGEK (*TpWRKY22*) and WIKYGQK (*TpWRKY11*). In soybean, *GmWRKY6* and *GmWRKY21* have been mutated to the WRKYGKK motif, which results in their inability to bind W-box (Zhou et al., 2008). In tobacco, conserved structural domain of *NtWRKY12* is mutated to WRKYGKK and binds specifically to the WK box (TTTTCCAC) (van Verk et al., 2008). Consequently, our hypothesis suggests that mutations in the WRKYGQK sequence could lead to a substantial reduction or even a complete loss of binding capacity to the W-box. Alternatively, it may result in binding to other motifs, thus participating in novel regulatory mechanisms.

Throughout the evolution history of the plant *WRKY* gene family, group I served as the ancestral group for group II and group III. The further evolution of the *WRKY* gene family led to the formation of group II and group III, involving the retention and deletion of the WRKY structural domain at the N-terminus of group I, as well as modifications in the Zinc finger structural domain at the C-terminus (Wu et al., 2005; Zhang and Wang, 2005). The phylogenetic analysis of WRKY sequences in *Arabidopsis* and rice combined the four branches of clade II into two new clades: IIa + b and IId + e (Rinerson et al., 2015). This may imply that there is a close evolutionary relationship between them, with genes in group IIa being closely related to genes in group IIb, and genes in group IId being closely related to genes in group IIe(Javed and Gao, 2023). These results may shed light on the function of unknown TpWRKYs in red clover based on identified functions of AtWRKYs in *Arabidopsis. TpWRKY56* in Group II-a subfamily may have similar functions to *AtWRKY18*, *AtWRKY40*, and *AtWRKY60* and may be involved in plant defense in response to ABA and drought stress (Chen et al., 2010). *AtWRKY32* promotes photomorphogenesis development through the COP1-HY5 signaling pathway. Under dark conditions, COP1 exerts negative regulation over the stability of *WRKY32*. Additionally, *WRKY32* directly interacts with the promoter of HY5, leading to the activation of its transcription and consequently promoting photomorphogenesis (Zhou et al., 2022). This suggests that *TpWRKY8* in *T.pratense* may also be associated with plant photomorphogenesis.

Examining the exon-intron structure of gene families aids in the identification of diverse structural domains and can serve as crucial evidence for discerning the evolutionary relationships of genes (Wang et al., 2011; Gorlova et al., 2014). In this research, a comprehensive analysis of the structural distribution of *WRKY* family members was conducted and discovered that *WRKY* genes within the same subfamily exhibit analogous structural distribution patterns. Three members of each the 11 *TpWRKYs* gene within group I have three, four and five introns, while the other two members have six introns. Members in subgroup IIa all contain three introns, and 83% of *TpWRKYs* have two introns in subgroup III (**Figure 2**), which is similar to the distribution in *Scutellaria baicalensis* and rice (Xie et al., 2005; Zhang et al., 2022a). The results of the analysis indicate that distribution patterns of exons and introns were closely related to the subfamily classification. The conserved features of motifs reveal that motif1, 2, 3, 4, and 7 are typical in group I, motif1, 2, 3 are typical in group II, and motif1, 2, 3, and 4 are typical in group III, which is more similar to the motif distribution observed in walnut (Hao et al., 2021). Almost all proteins contained motif1, motif2 and motif3 which is further evidence that all proteins contain conserved WRKY structural domains. Compared to alfalfa, chickpea, and common bean, structural domains 1 and 2 embody the characteristic features of WRKY DNA-binding domains, remaining entirely conserved across all WRKYs, with the exception of a few domains that may be missing (Wang et al., 2016; Waqas et al., 2019). On the other hand, abundant cis-acting elements were found in the upstream 2 kb of the red clover *WRKY* genes, which can be classified into four categories (development-related, phytohormone responsive, stress-responsive and light-responsive elements) and are associated with ABA, JA, SA and multiple biotic/abiotic stresses. These cis-acting elements of *TpWRKYs* gene bind to various stress-related trans-acting factors to regulate the expression and response of red clover stress resistance genes.

Replication modes of genes include whole genome duplication (WGD), tandem duplication, segmental duplication and transposon-mediated transposon duplication, which are the major modes of eukaryotic genome evolution (Panchy et al., 2016). The expansion of the *WRKY* gene family is predominantly driven by tandem and segmental duplication events (Rushton et al., 2010). In sunflower (*Helianthus annuus* L), 21 *HaWRKY* genes were identified as resulting from segmental duplication events on 10 chromosomes, as well as six tandemly duplicated gene pairs found on chromosome 17 (Guo et al., 2019). In pineapple (*Ananas comosus*), seven tandem repeat gene pairs were identified within the *WRKY* gene family, with 17 fragment duplication events in 27 genes (Xie et al., 2018). In maize (*Zea mays*), 52 gene pairs out of 125 *ZmWRKY* genes were identified to be involved in segmental replication events and no tandem replication events were found (Hu et al., 2021). We hypothesize that tandem and fragment replication events promote the expansion of the *TpWRKY* gene family. There are eight pairs of synteny segments within the red clover. Furthermore, we observed that genes with tandem repeat events belong to the same subfamily, such as *TpWRKY5* and *6* belonging to IIa, *TpWRKY30* and *31* belonging to I, and *TpWRKY54* and *55* belonging to IIe. Meanwhile, the synteny map of *TpWRKY* family with monocotyledons (*Oryza sativa*) and dicotyledons (*Arabidopsis thaliana*) was also constructed. These results showed that there were 17 synteny pairs in red clover and *Arabidopsis*, and three pairs of synteny gene pairs with rice. The lower number of synteny gene pairs between red clover and monocotyledons suggested that these gene pairs likely emerged after the divergence of dicotyledons and monocotyledons.

The interconnection between gene expression and gene function is widely acknowledged. Investigating the expression patterns of genes in various tissues is of paramount importance for mining functional genes (Dutta et al., 2018). Research has demonstrated that *WRKY* genes exhibit expression in one or more tissues, and they are crucial for plant growth and development (Feng et al., 2022; Zhang et al., 2022b). The evolutionary relationship between *CsWRKY7* and *AtWRKY7* in *Camellia sinensis* is closely related, and root elongation was higher in *CsWRKY7* than in wild type (Chen et al., 2019). Under stress conditions, seedlings of *WRKY11* and *17* knockout mutants in *Arabidopsis thaliana* exhibited reduced germination rates and impaired root growth compared to the wild type (Ali et al., 2018). We analyzed the transcript levels of red clover *WRKY* genes in roots, stems, leaves, and flowers by RNA-Seq. These genes were expressed at the highest level in the root system (**Figure 6**), such as *TpWRKY9*, *TpWRKY19*, and *TpWRKY46*, and were more closely related to *AtWRKY7*, *AtWRKY11*, and *AtWRKY17* evolutionarily in *Arabidopsis* (**Figure 1**). These genes potentially hold significant part in the growth and development of red clover roots and could serve as potential candidates for further functional studies related to the root system.

WRKY proteins play transcriptional regulatory roles in plant adaptations to various stress environments (Wani et al., 2021). In rice, *Arabidopsis* transformed by *OsWRKY45* increased drought resistance by regulating stomatal closure and stress-related genes (Qiu and Yu, 2009). A novel type WRKY TF, *DgWRKY5*, isolated in *Chrysanthemum* was up-regulated under salt, ABA and H_2_O_2_ (Liang et al., 2017). WRKY TFs play essential roles in plant adaptation to abiotic stresses, and these adaptations are the consequences of the interaction between *WRKY* genes and multiple plant hormones. It has been shown that exogenous administration of different plant hormones can alter the expression of *WRKY* genes under various abiotic stresses such as cold and salt (Gulzar et al., 2021; Huang et al., 2021). WRKY TFs work synergistically or independently in response to diverse stresses. For instance, ABA-induced synergism between two rice *WRKY* genes (*OsWRKY51* and *OsWRKY71*) suppressed gibberellin (GA) signaling in embryos and aleurone cells (Xie et al., 2006). In *Arabidopsis*, *AtWRKY18*, *40*, and *60* are involved in signaling pathways mediated by the phytohormones SA, JA, and ABA (Chen et al., 2010). As previously described, a considerable number of cis-acting elements related to stress and hormone responses were identified in the *WRKY* genes of red clover. Of the 59 *TpWRKY* genes, 46 contained ABRE, 43 contained CGTCA-motif, 45 contained ARE, 24 contained MBS, and 20 contained LTR. This suggests that *TpWRKY* genes play a role in regulating a diverse range of plant hormone and stress response pathways.

During this research endeavor, we examined the expression levels of 15 selected genes under drought, low temperature, MeJA and ABA treatments by using RT-qPCR. The findings indicated that drought stress induced a significant up-regulation of *TpWRKY46* (**Figure 7A**). *TpWRKY46* contains two drought cis-acting response elements (MBS). Cold stress significantly induced the expression of *TpWRKY26* and *TpWRKY41* (**Figure 7B**). Interestingly, these two genes do not have cis-acting elements related to low temperature, and we hypothesize that there may be other factors or regulatory pathways besides low-temperature induction that would lead to the high expression of these two genes under low-temperature conditions, as well as the possibility that these two genes may be subjected to subsequent regulation after low-temperature induction, e.g., post-transcriptional regulation, post-translational modification. Among all genes tested, *TpWRKY29* and *TpWRKY36* were the most highly expressed under MeJA stress (**Figure 7C**), *TpWRKY17* and *TpWRKY46* were the most highly expressed under ABA stress (**Figure 7D**). *TpWRKY29/36* contained six and four MeJA response elements, respectively, and *TpWRKY17/46* both contained two ABA response elements. This suggests that these cis-acting elements may play a regulatory role under various stresses and provide candidate genes for the selection of stress resistance genes in red clover, which will be further functionally verified in subsequent experiments.

## 5 Conclusion

In this research, we conducted a comprehensive genome-wide identification of *WRKY* gene family members in red clover, leading to the identification of a total of 59 *TpWRKY* genes. According to the phylogenetic relationships, these genes were categorized into three groups, and within the second group, further subdivision into five subgroups was observed. The physicochemical properties, phylogeny, gene structure, conserved motifs, cis-acting elements, and collinearity of these WRKY proteins were analyzed to establish a foundation for comprehending the evolutionary relationships of the *TpWRKY* gene family. In addition, we explored the expression patterns of *TpWRKY* genes in different tissues by RNA-seq and RT-qPCR, and selected *TpWRKY17*, *TpWRKY26*, *TpWRKY36* and *TpWRKY46* among 12 *TpWRKY* genes, which were highly induced after being subjected to abiotic stresses. This study reveals the basic characteristics of the *TpWRKY* gene family, which lays the foundation for the excavation of resistance genes in red clover and promotes the breeding and propagation of red clover.

## Data availability statement

The original contributions presented in the study are included in the article/**Supplementary Material**. Further inquiries can be directed to the corresponding authors.

## Author contributions

GY: Conceptualization, Data curation, Formal analysis, Investigation, Software, Validation, Visualization, Writing – original draft, Writing – review & editing. NZ: Investigation, Software, Validation, Writing – review & editing. YZ: Writing – review & editing, Validation, Visualization. YH: Writing – review & editing, Data curation, Software. JP: Writing – review & editing, Software, Validation. YL: Data curation, Software, Writing – review & editing. WZ: Conceptualization, Funding acquisition, Methodology, Resources, Supervision, Writing – review & editing. BL: Conceptualization, Funding acquisition, Methodology, Resources, Supervision, Writing – review & editing.
