# Supplementary material for: Genome-wide identification and expression analysis of WRKY gene family members in red clover (Trifolium pratense L.)
Source: Front Plant Sci. 2023 Dec 7;14:1289507. doi: 10.3389/fpls.2023.1289507 (PMC10733489; doi:10.3389/fpls.2023.1289507)
Supplement: Supplementary file 1 [file DataSheet_1.pdf]

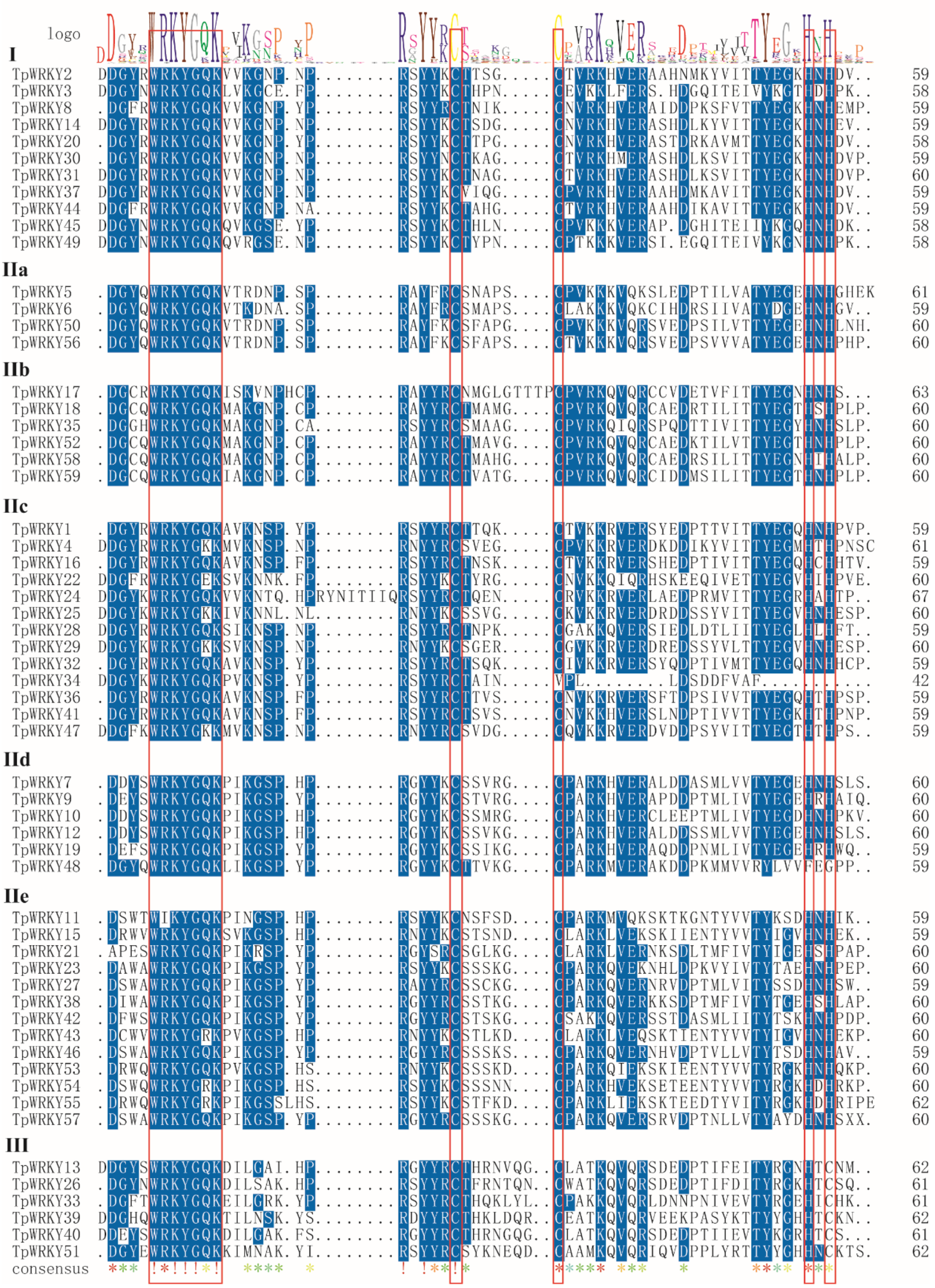

Figure S1. Multiple sequence alignment of the WRKY domain from TpWRKYs. Blue indicates conserved amino acid residues.
