# Supplementary material for: Genome-wide identification and expression analysis of WRKY gene family members in red clover (Trifolium pratense L.)
Source: Front Plant Sci. 2023 Dec 7;14:1289507. doi: 10.3389/fpls.2023.1289507 (PMC10733489; doi:10.3389/fpls.2023.1289507)
Supplement: Supplementary file 2 [file DataSheet_2.pdf]

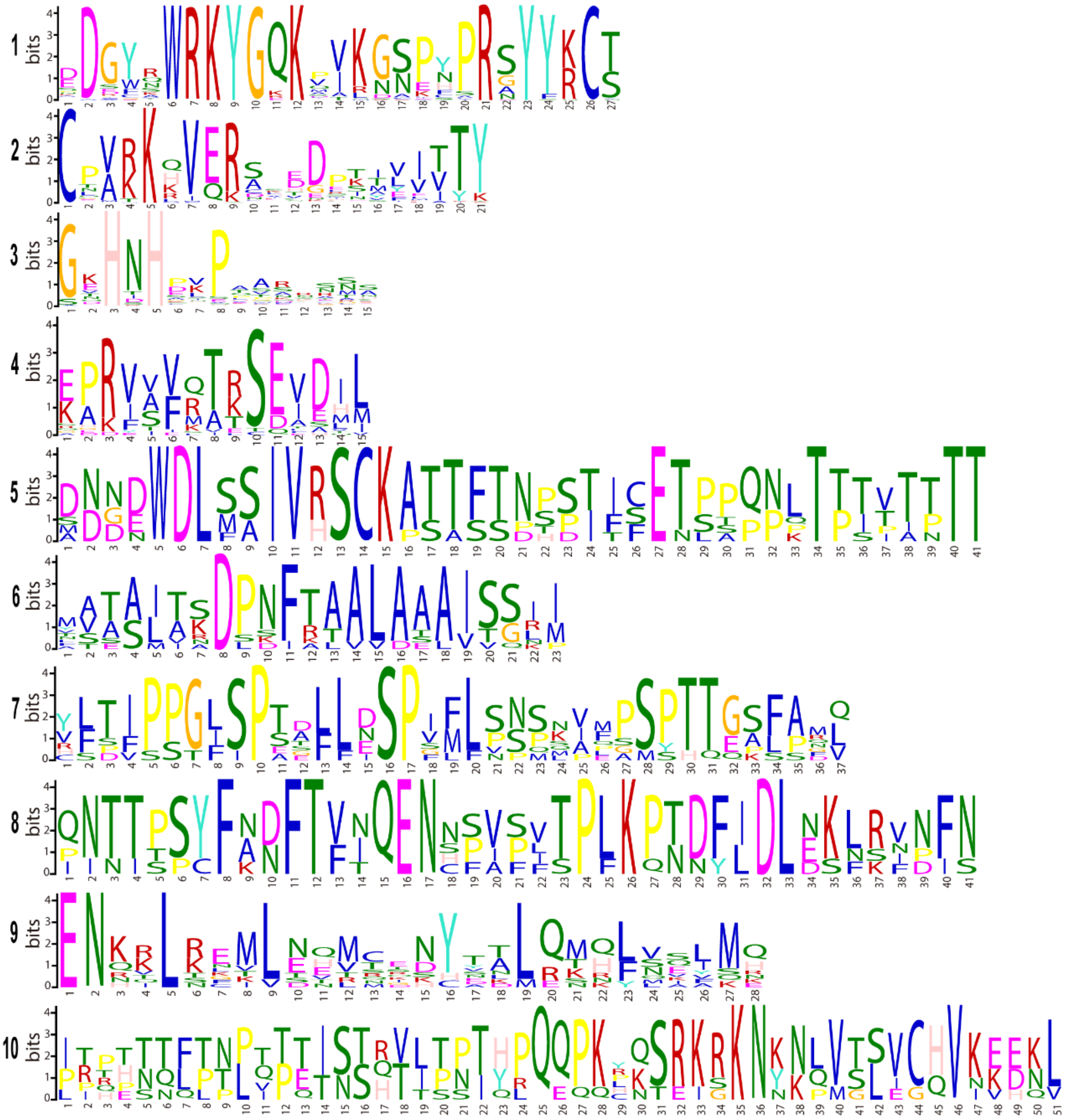

Figure S2. Sequence logo of the TpWRKY proteins motifs. The height of each amino acid represents the relative frequency of the amino acid at that position.
